# Efficacy of an internet-based self-management intervention for depression or dysthymia – a study protocol of an RCT using an active control condition

**DOI:** 10.1186/s12888-019-2063-1

**Published:** 2019-03-14

**Authors:** Caroline Oehler, Frauke Görges, Daniel Böttger, Juliane Hug, Nicole Koburger, Elisabeth Kohls, Christine Rummel-Kluge

**Affiliations:** 1 German Depression Foundation, Semmelweisstr. 10, 04103 Leipzig, Germany; 2 European Alliance Against Depression, Semmelweisstr. 10, 04103 Leipzig, Germany; 3 Research Academy Leipzig, Wächterstraße 30, 04107 Leipzig, Germany; 4 Department of Psychiatry and Psychotherapy, Medical Faculty, University Leipzig, Semmelweisstr. 10, 04103 Leipzig, Germany

**Keywords:** Internet based intervention, IBI, IMI, iCBT, Online self-management, Depression, eMentalHealth, Study protocol, iFightDepression

## Abstract

**Background:**

The treatment of major depressive disorder, a highly prevalent disorder associated with pronounced burden, is a large challenge to healthcare systems worldwide. Internet based self-management interventions seem to be a cost effective way to complement the treatment of depressed patients, but the accumulating evidence is mainly based on the comparison to waitlist controls and treatment as usual, which might lead to an overestimation of effects. Furthermore, studies assessing long-term effects and possible negative outcomes are still rare.

**Methods/Design:**

The proposed study evaluates the efficacy of the German version of the iFightDepression® tool in comparison to an active control condition. A total of 360 patients with mild to moderate depressive symptoms are included into a two-armed randomized controlled trial. They receive one of two six week interventions; either the iFightDepression® tool or progressive muscle relaxation serving as the control condition. Both intervention groups receive information material, weekly tasks via the internet and regular phone calls as part of the intervention. The primary outcome is change in depressive symptoms after the intervention period, as measured with the Inventory of Depressive Symptomatology. Satisfaction with the program, usability, changes in perceived quality of life, and possible negative effects are assessed as secondary outcomes.

**Discussion:**

This study represents the first randomized controlled trial on the iFightDepression® self-management tool in its German version, aiming at efficacy, but also at providing new insights into so far understudied aspects of E-mental health programs, namely the specificity of the treatment effect compared to an active control condition, it’s continuity over a time course of 12 months, and possible negative effects of these internet based interventions.

**Trial registration:**

International trial-registration took place through the “international clinical trials registry platform” (WHO) with the secondary ID 080–15-09032015. German Clinical Trial Registration: DRKS00009323 (DRKS.de, registered on 25 February 2016).

## Background

Major depressive disorder (MDD) is highly prevalent and associated with pronounced psychological burden. In 2010, more than 74 million “healthy life years” were lost worldwide due to MDD and dysthymia [1]. The twelve-month prevalence in Europe was estimated to be at 6,9% for MDD [2]. In Germany, a point-prevalence of 8,1% for depressive symptoms was reported [3], resulting in reduced quality of life and a loss of 15–22 billion € for the national economy (calculated for 2008) due to reduced capability to work, presentism and other consequences of depression [4]. Even mild forms of depression are associated with significant reductions in quality of life and well-being [5].

While evidence-based treatments for depression are available (cognitive behavioral therapy (CBT) and pharmacotherapy [6]), not everyone affected by depression has access to these treatments or seeks help. Several reasons such as barriers in access to care and reluctance to seek help due to stigma are contributing to this situation [7]. If help is sought, there are a number of further factors affecting the quality of care. For instance, treatment adequacy has been found to be especially low in primary care [8]. This affects a large number of patients (62,5%), who often receive treatment from their general practitioner (GP) after being diagnosed with depression [9].

One promising approach to improve the accessibility and quality of treatment is to complement it by evidence-based self-management programs delivered online. Through the internet, it is possible to offer low-threshold interventions to a large number of people independent of time and place [10]. As people suffering from depression do not differ in their use of the internet from the general public and more than 80% of patients had used the internet for researching health information [11, 12], the internet is suitable to provide support for depressed patients.

Evidence supporting the effectiveness of online interventions has grown rapidly over the past years. In the treatment of depression, internet based interventions (IBIs), most often in the form of cognitive behavioral therapy (iCBT), have been found to be effective compared to both waitlists and treatment as usual (TAU), when combined with personal contact, email or telephone support [13, 14]. It has been shown that online interventions with at least a minimum amount of guidance show larger effect sizes and higher completer rates compared to unguided interventions [15]. Further evidence suggests that IBIs could be a cost-effective adjunct to the treatment of depression [16, 17]. A recent meta-analysis, comparing IBIs with face-to-face psychotherapy for depression, yielded similar effect sizes for both approaches [18], although, due to small sample sizes, it is questionable whether the meta-analysis was adequately powered to detect inferiority.

Although evidence is accumulating quickly, several important aspects of IBIs have not yet been studied sufficiently, or at all:

First, little is known about possible adverse effects of internet-based interventions for depression. While symptom deterioration in RCTs on internet-interventions is comparable to psychotherapy studies [19], and occur less often in the intervention group compared to the control conditions [20], other possible undesired effects such as disappointment or feeling dependent on the intervention are still lacking empirical research. A systematic assessment of possible deterioration and especially other possible adverse effects is needed [21] and will be targeted in the present study, to expand the understanding of negative effects beyond symptom deterioration.

A second area of IBIs that, to our knowledge, has not been studied yet, is the “specificity” of IBIs. The term “specificity” has been used in psychotherapy research in contrast to unspecific treatment effects (e.g. contact to a healthcare professional, a treatment procedure or “ritual” that is perceived as helpful by the patients). Specific effects are provoked by the applied therapeutic methods (e.g. cognitive restructuring, hypothesis testing) [22]. According to Baskin and colleagues, a credible control condition should match the intervention with regard to the number of sessions, the treatment modality and the profession and experience of the therapist delivering the intervention [23], in order to achieve a comparable level of unspecific effects of the intervention. So far, most trials investigating IBIs have mostly compared it either to treatment as usual (TAU) or to waitlist controls (WL) [13]. It has been shown that the choice of control condition contributes largely to the effect sizes of RCTs with comparisons to treatment as usual (TAU) or to waitlist controls (WL) producing the largest effect sizes [24]. In a meta-analysis on RCTs, Papakostas and Fava found that the probability of receiving placebo vs. an active treatment influenced the size of the placebo effect in patients suffering from depression [25]. This underlines the impact of expectation of success in the treatment of MDD. Some authors argue that trials using WL as a control condition are especially inept for depressed patients because being randomized into a waiting group might lead to less help seeking behavior than a no treatment or TAU control and therefore might even have a negative effect on the symptoms [24, 26].

Further, it is evident, that in the treatment of depression the expectation to receive a treatment (placebo) in itself contributes largely to the treatment effect [27]. Most probably, this is also true for trials investigating IBIs. To separate specific effects of a psychological intervention from expectation/placebo effects, it is favorable to compare with an active control similar in duration, induction of hope and the contact with the researchers. For the current trial, progressive muscle relaxation was used as an active control condition. Relaxation is also highly accepted by the public as a form of self-help for depression [28, 29] and rated as helpful by clinically depressed patients [30]. PMR itself was reported to elicit small antidepressant effects superior to waitlists, when practiced on a regular basis and mainly in group settings, but significantly less effective than psychotherapy [31] and should yield even smaller effects with reduced personal contact in its online version. Although not completely inert as a placebo pill could be, its high credibility and usability paired with limited antidepressant effects make it a good choice as an active comparator. By providing the option of an active control condition, the current study will be able to obtain an estimate of the difference of unspecific effects of online supported self-management interventions and an iCBT program that was specifically designed to teach skills for coping with depression.

Additionally, very few studies have investigated long-term effects of IBIs so far, as stated in the meta-analysis by Andersson and Cuijpers [32]. The few studies reporting long-term results yield widely differing results. Andersson et al. [33] found large within-group effect sizes of an IBI for depression after a three year follow-up and superior results compared to group therapy, but the results cannot be clearly separated from spontaneous remission since no control condition was evaluated. On the other hand, Eriksson et al. [34] reported no additional effects of an IBI when compared to treatment as usual for primary care patients after 12 months. A long-term comparison against an active control condition could help to clarify what amount of symptom reduction over time can be attributed to the techniques taught in IBIs.

The current study’s goal is to fill some of the gaps in the current literature body on iCBT and to extend the evidence base for online interventions. It investigates the following objectives by evaluating the German version of the iFightDepression® tool (iFD®), a free-to-use, multilingual, guided online self-management tool that is based on the principles of cognitive behavioral therapy.

The primary objective of the study is to investigate the efficacy of the iFD® tool regarding changes in symptom severity compared to an active control group using progressive muscle relaxation (PMR) during a six week intervention period and at the follow-up assessments after 3, 6 and 12 months.

Based on the previous results on the efficacy of IBIs, we hypothesize that the iFD® tool will be superior compared to PMR in reducing depressive symptoms directly after the six weeks of intervention, and at follow-up.

The secondary objective is to examine possible negative effects, usability and satisfaction with the program and potential changes in health related quality of life (all assessed during the intervention period and at the follow-up assessments).

We hypothesize, that the iFD® tool will lead to a significantly greater improvement on quality of life, especially concerning mental well-being, than PMR and that both interventions will be equally usable and satisfactory for the users. Possible negative effects and subgroup differences will be examined in an explorative manner.

## Methods

### Study design

**Fig. 1:**
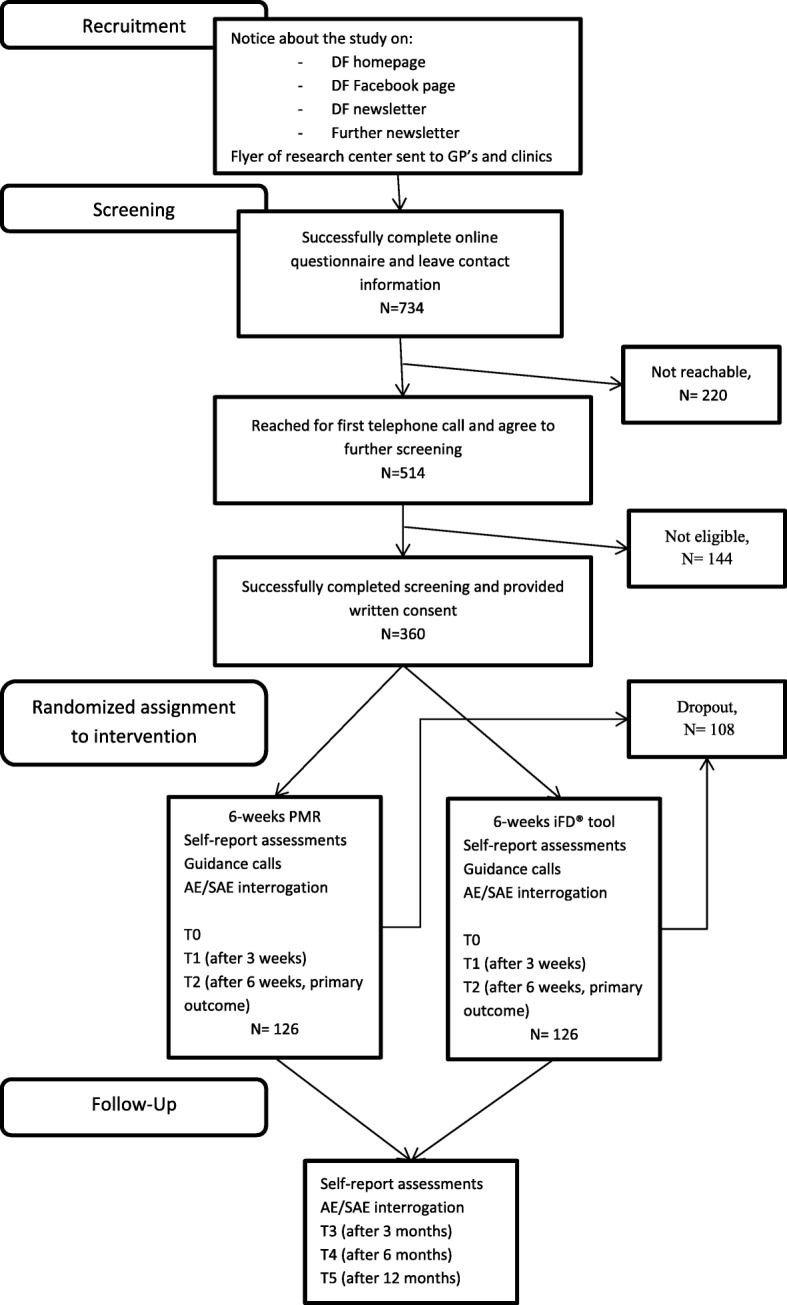
Participant flow, the numbers provided are the estimates based on the power analysis and dropout percentages in previous studies [35]. (DF = depression foundation, AE = adverse event, SAE = serious adverse event, PMR = progressive muscle relaxation, iFD® tool = iFightDepression® tool)

A two-arm randomized trial is conducted to compare efficacy and usability of a guided online-self-management intervention (iFightDepression®) with an active control condition (PMR-Training) after the six weeks of intervention and at 3-, 6- and 12-month follow-up. Figure 1 summarizes the process of recruitment, screening, randomization and measurement points.

Power analysis to determine sample size:

For the power calculation, results of the study by Hegerl et al. were used to estimate the expected difference for the primary outcome between intervention and active control group in the present study [35]. The study compared CBT to an active control condition (guided self-help group) and found a difference of 5.3 points and a pooled standard deviation of 11.1 on the IDS-C after 10 weeks. Since the present intervention takes only 6 weeks and the effect sizes might generally be smaller for online self-management compared to face-to-face treatment, we expect a difference of 4 points on the IDS-SR. The calculation was performed in G*Power (3.1.9.2) using the power analysis for independent groups. To detect this effect with a power of 80% (alpha = .05), the number of cases needed per group is 122. Considering the expected dropouts, this trial is powered for completers.

### Recruitment and study settings

The study is a fully remote RCT and participants are recruited throughout Germany. Different recruitment channels are used: The study is promoted primarily by the German Depression Foundation (DF) via newsletter (approx. 10.000 recipients), the website, the Facebook page and print flyers sent to GPs (for details on the participant flow, see Fig. 1). Furthermore, newsletters of associated organizations are used for distribution. Recruiting through different channels provides a wide reach, hopefully leading to an unbiased sample of people open to use online-self-management. With this recruitment strategy, the study aims at analyzing a sample consisting of patients searching either online help or seeking help from their GPs.

### Eligibility criteria

Inclusion and exclusion criteria can be found in Table 1. They are assessed at baseline, first through an online screening questionnaire (filled out by the patient) and a telephone interview (details are shown below).

**Table 1:** Inclusion and exclusion criteria

Inclusion criteria	
1. Written informed consent	
2. Age ≥ 18 years	
3. Outpatient status	
4. Mild or moderate depression according to PHQ-9 at the time of screening	
5. Past or present diagnosis of depression or dysthymia according to MINI	
6. Sufficient language skills (regarding both speaking and writing)	
7. Internet-access (privately at home or sufficient opportunity to access the internet regularly)	
Exclusion criteria	
1. ICD-10 diagnoses: dementia, alcohol/ drug addiction; schizophrenia; manic episode or bipolar disorder; obsessive-compulsive disorder according to MINI	
2. Known personality disorder (F60.2, F60.31)	
3. Acute suicidal tendencies	
4. Severe somatic disorders requiring immediate treatment	
5. Participation in another clinical trial within the last 4 weeks	
6. Known alcohol/ drug abuse within the last 6 months	
7. Pregnancy	

Eligibility criteria are meant to select a sample that is similar to the target population of the iFightDepression® tool, i.e. patients with milder forms of depression in primary care. Since the tool is designed as a supplement to improve standard treatment, participants are required to already receive treatment by a physician or a licensed psychotherapist.

### Screening and consent procedure

Participants who are interested in the study are directed to an online pre-screening tool, which contains information about the study and a questionnaire to be filled out by the patient to assess the following initial in- and exclusion criteria: minimum age, outpatient status, internet-access, no pregnancy, no severe somatic disorder requiring immediate treatment, no trial participation within the last 4 weeks and no known personality disorder, as well as depression severity (assessed using the Patient Health Questionnaire, PHQ-9). Those fitting the criteria (see above), obtaining a score of 5–14 on the PHQ-9 (indicating mild or moderate depression) and indicating their interest to participate in the study are asked to leave their contact information to be called by a study assistant. During a first telephone contact, the participant is informed about the study’s objectives and procedures and, if still interested, a telephone interview for in depth assessment is arranged.

During the telephone interview, sociodemographic data, language skills, acute suicidal tendencies, current depression severity by PHQ-9, severe somatic disorders and medical history are assessed. A clinical interview (M.I.N.I.) is conducted to confirm eligibility for the study and to explore potential ICD-10 diagnoses that would require exclusion. At the same time, the M.I.N.I. is used to confirm the depression diagnosis (former or present).

Participants who fulfil the eligibility criteria are informed about the study design and asked to send a written informed consent via fax or regular mail. After receiving the signed informed consent at the study site, the participant is enrolled in the study. If acute suicidal risk is reported (indicated by either reporting having suicidal thoughts on several days of the week in the PHQ, or according to the section on suicidality in the M.I.N.I.) during the screening process, it is assessed using a well-established procedure to ensure appropriate clinical support.

### Randomization and allocation

All eligible participants who provide written informed consent are randomly assigned to one of the two intervention arms (see also Fig. 1). The randomization is carried out weekly by the Center for Clinical Trials (University Leipzig) using a self-developed software, using the minimization algorithm by Pocock [36] stratified for gender (male/female), depression severity (mild/moderate according to PHQ-9 during screening), and CBT-experience (present/absent) with an 80% chance using the algorithm’s recommendation to yield well-balanced groups. This procedure guarantees to keep allocation concealed for the study assistants. After allocation, blinding is not possible, because study assistants also act as “guides” for the interventions during the study. We do not consider this a risk of bias, as the results are based on self-ratings only.

### Interventions

#### The iFightDepression® tool (iFD® tool)

The guided iFD® self-management tool was developed based on CBT, on best practice examples and internationally consented within the EU-funded project Predi-Nu [37]. It is currently available in 11 languages. It includes six core workshops (see Table 2), each comprising written information, worksheets, exercises and a mood rating. For study purposes, patients are asked to use the tool for six weeks and complete one workshop per week.

**Table 2:** Description of the six core workshops of the iFightDepression® tool

Workshop	CBT-based Content
Thinking, feeling and doing	- Participants receive information about how thoughts, feelings and behavior are interconnected. - The model of depression as a “downward spiral” is introduced. - Documentation of daily activities and corresponding mood changes is encouraged to identify ways to improve daily routines. Task: Activity diary
Sleep and depression	- The possible connection between long bedtimes and worsening of mood is described. - Participants are guided to explore whether there is a connection between their bedtimes and changes in mood. Task: Sleep diary
Planning and doing enjoyable things	- Users are instructed to plan ahead and to integrate at least one positive activity into their daily routines. - Findings of the first week are used and expanded in this workshop. Task: Plan one week ahead including at least one positive activity a day
Getting things done	- Focus on training problem solving abilities - The tool user chooses one problem they want to tackle and breaks it down into small steps that are realistic and achievable. - Solutions for possible barriers or difficulties are to be anticipated. Task: Break down one task into small steps and plan ahead when and how to complete it
Identifying negative thoughts	- The “ABC model” is introduced, participants learn about unhelpful thoughts. Task: Identify negative thoughts during one event that made one feel bad
Changing negative thoughts	- Participants are briefed to look for the cause of negative thoughts and find possible and helpful alternatives. Task: Develop alternative thoughts

Participants are encouraged to work with the tool on a regular basis. Each week, they are asked to read the information provided in the respective workshop and to use the corresponding exercise for at least one week. Participants can choose to complete worksheets online or to use a printed version if they find it more convenient.

The iFD® tool also incorporates a mood rating (PHQ-9, German version) with a graphical output display. It can be used as often as desired, but is mandatory once a week.

The iFD® tool was developed as a guided program; it was designed for complementing depression treatment in routine care. In the context of routine care, guidance is offered by physicians and licensed psychotherapists. Guides are required to complete a guidance webinar or face to face training and a short test before gaining access to the tool. They are instructed to focus on administrative and motivational support. Guides are motivating and positively amplifying participants and explore how well participants were able to integrate the practice into their daily routine.

For this study, the guides are trained psychologists and psychotherapists from the Research Centre of the German Depression Foundation. They have qualified through the webinar, are required to have a degree in psychology and are supervised by a senior psychiatrist who was involved in the development of the iFD® tool.

#### Progressive muscle relaxation (PMR)

In the present study, progressive muscle relaxation (PMR) is used as an active control condition. It was chosen because it is widely used in therapeutic settings, e.g. as part of cognitive behavioral therapy and in the treatment of sleep disorders. PMR is also highly accepted by the public as a form of self-help for depression [28, 29] and rated as helpful by clinically depressed patients (38% very/moderately effective, 40% slightly effective [30]). In a systematic review, relaxation (PMR or similar techniques) was recommended as first line treatment in a stepped care approach. Antidepressant effects were visible shortly after relaxation interventions, superior to waitlist and no treatment, but inferior to psychotherapy [31].

The mechanism behind PMR was first described by Jacobson in 1934. He developed the concept to induce mental relaxation through relaxing the body. Muscle groups are tightened and then relaxed with the attention of the patient focused on the contrast between tension and relaxation. Through regular practice, a sensitization to tension in the body takes place and relaxation can be induced at will [38].

In the present study, PMR is used as the control condition. During the six weeks of training, participants are encouraged to practice PMR and learn how to deliberately induce physical relaxation to reduce stress and mental tension. Lessons range from 13 to 33 min and build on one another, adding more muscle groups every week. At the beginning of each week, participants receive a link to download the next lesson. They are instructed to practice on a daily basis, if possible, but at least two or three times a week and to integrate the practice into their daily routine.

#### Guidance

To keep the groups comparable with respect to the contact with the study staff, both groups receive the same amount of guidance and are guided by the same psychologists and psychotherapists.

During the intervention period, participants are called five times. Three of these calls are also used for assessment (see below), and two calls are pure guidance calls. Participants get the chance to ask questions regarding the tasks of the previous weeks. Guides follow a guidance protocol that was developed based on the guide manual for the iFD® tool in routine care. They are instructed to focus on motivational and clarifying questions and calls are planned not to exceed 20 min. Length and content of the call are noted and the perceived quality is rated by the study guides. These procedures apply for both groups, treatment and active control, to allow for a check for equivalence.

After the six week study period, participants are free to continue using the respective intervention on their own.

### Assessment

Assessments take place just before the onset of the intervention period (T0), three weeks after the intervention has started (T1) and at the end of the intervention (six weeks after start of the intervention; T2). Follow-up surveys take place 3, 6 and 12 months after the end of the intervention (T3-T5; see Table 2 for a detailed overview and the measures used at each time point). Each assessment consists of an online questionnaire and a telephone interview. The PHQ-9 is filled out weekly for monitoring purposes. The access information to the online questionnaires is e-mailed to participants automatically by the Center for Clinical Trials, at the corresponding dates after inclusion, using secure links.

### Description of outcome measures and instruments

#### Screening

The Mini International Neuropsychiatric Interview (M.I.N.I. 5.0.0) is used as a diagnostic instrument. It is a short diagnostic structured interview, used in its clinician rated version (M.I.N.I.-CR) to assess 14 psychiatric disorders according to DSM-IV. It is a valid and reliable instrument that can be applied in a reasonable amount of time with sensitivity scores ranging from .46 to .94, specificity scores ranging from .72 to .97 and a good to very good concordance between M.I.N.I. and Composite International Diagnostic Interview (CIDI), which represents the gold standard (e.g. kappa = .73 for major depressive episode [39] see [40] for a comparison to SKID).

#### Primary outcome

Primary outcome of this study is the reduction of depressive symptoms in the treatment group compared to the control condition as measured by the Inventory of Depressive Symptomatology score (IDS-SR) after the 6-week intervention. The IDS-SR is used in its German, self-rating version. The concordant validity of the German version with the Beck Depression Inventory and the Hamilton Rating Scale for Depression has been shown to be good (r > =.88) [41]. Furthermore, the scale has been shown to be useful in detecting symptom change as well as residual symptoms in depressed patients [42].

#### Secondary outcome

Secondary outcomes in this study are changes in depressive symptoms over the course of the intervention, acceptance (satisfaction with) and feasibility of the iFightDepression® tool, changes in perceived quality of life and adverse treatment effects (see Table 3).

**Table 3:** Overview of assessment points and measurements used

Measurement time	Assessment													
	In−/exclusion criteria	Verbal consent	Sociodemographic data	Medical history, medication, therapies	M.I.N.I	PHQ-9 (Patient Health Questionnaire)	Written consent	IDS (Inventory of Depressive Symptomatology)	Content and subjective quality of guidance	Documentation AE/SAE	Evaluation of workshop/program + frequency of use	SF-12 (health related life-satisfaction)	INEP (assessment of side effects, adapted; modified for online usage)	CSQ-8 (patient satisfaction, adapted)
Online screening	P					P								
Telephone interview		P	SA	SA	SA	SA	P							
T0						P		P	SA	SA		P		
Week 1						P			SA		P			
Week 2						P					P			
T1						P		P	SA	SA	P	P	P	
Week 4						P			SA		P			
Week 5						P					P			
T2						P		P	SA	SA	P	P	P	P
T3–3 months						P		P		SA		P	P	
T4–6 months						P		P		SA		P	P	
T5–12 months						P		P		SA		P	P	

(P = patient, SA = study assistant)

Changes in depressive symptoms over the course of the intervention are measured with the nine-item Patient Health Questionnaire (PHQ-9). The PHQ-9 is a short, well validated and widely used measure, of which validity and sensitivity to change have been shown repeatedly [43, 44].

The acceptance and feasibility of the iFightDepression® tool is measured using the client satisfaction questionnaire (CSQ-8) in its German version (ZUF-8). Originally developed to measure client satisfaction with a therapy or service, an adapted version of the CSQ-8 is used to assess user satisfaction in the present study. The wording of some of the items has been adapted slightly to fit the context of web-based interventions. The internal consistency of a similar adaptation has been shown to be good (omega = .95) and ratings of satisfaction correlated with symptom reduction [45]. Four extra items, specifically designed for participants to rate different aspects of each workshop, were added. Participants also state whether they felt better after completing the last workshop and whether they attribute the change to the intervention or to other factors. Additional items at post-treatment ask participants to rate the entire intervention.

Changes in perceived quality of life are rated using the SF-12. To provide a practical short form of the SF-36, the SF-12 was developed to measure health related quality of life. The SF-12 consists of a mental and a physical component score. Its moderate to high convergent validity has been shown in several studies [46, 47].

To monitor and measure adverse treatment effects, an adapted version of the INEP (Inventory for the assessment of negative effects of psychotherapy, [48]) is used. As the original version is designed to measure adverse effects of psychotherapy, the wording of the items was changed to fit the context of IBIs. Three Items, which were not transferable, were omitted. It is a relatively new measure consisting of 18 items covering different domains (e.g. symptom deterioration, interpersonal worries or decreased compliance with other therapies). For each adverse effect, participants rate whether it was caused by the intervention or not. Adverse events (all forms of symptom deterioration or other medical conditions needing to be treated) are assessed by the study assistants during the calls using a standardized protocol. Possible association to the intervention is rated.

### Quality assurance and data management

The study is designed in accordance with the declaration of Helsinki [49]. The study development and implementation is annually supervised by a scientific international expert advisory board and adapted towards their recommendations.

Procedures for data collection have been implemented by the Center for Clinical Trials at the University of Leipzig. Research data is collected in a pseudonymized manner by the Center for Clinical Trials. Data collection for online self-report measures as well as protocols completed by study assistants are centrally administered using LimeSurvey. The questionnaires are programmed in a way that all items have to be answered. The data are stored by the Center for Clinical Trials and will be provided to the research team upon request after the last patient finishes the intervention period and at the end of the data assessment period. Regular data-backups are performed.

Phone calls as described above are carried out to improve compliance with the interventions and online self-report questionnaires are installed to measure frequency and amount of intervention use. To keep the study procedures parallel across different study assistants, Standard Operating Procedures (SOPs) were developed and all study assistants are trained on the SOPs. Every Monday the study assistants receive feedback on the participants having filled out the questionnaires including the PHQ-9 sum score to check for deterioration or suicidality. At follow-up, patients are called again and, if necessary, reminded via email to fill out the respective questionnaire to assess possible adverse events during the follow-up period and to improve completeness of data. Patients reporting acute suicidality (score > 1 on the suicidality item of the PHQ) once or symptoms indicating severe depression (PHQ < 14) for three weeks in a row in the weekly questionnaires, or acute suicidality during the follow-up period are contacted using a well-established procedure to ensure appropriate clinical support.

#### Trial status and review

The trial was registered at the German Register for Clinical Trials (DRKS) under the title “Efficacy of an internet-based self-management intervention for adult primary care patients with mild and moderate depression or dysthymia”, identification code: DRKS00009323 on 2016-02-25. International trial-registration took place through the “international clinical trials registry platform” (WHO) with the secondary ID 080–15-09032015.

This trial was reviewed and approved by the ethics committee of the Medical Faculty, University of Leipzig on 2015-03-18.

Recruitment for the trial commenced in June 2016 and was finished in August 2018.

### Statistical analysis plan

Statistical reporting will follow the CONSORT standards [50].

For the main analysis, the mixed models approach with time and intervention group as fixed factors will be used to analyze whether there is a statistically significant difference between iFightDepression® and PMR concerning depressive symptoms after the 6-week study period, measured using the IDS-SR. A random intercept and a random slope for each participant will be added if beneficial for the model fit, with the subjects being nested within groups. This method is recommended in a scenario, where repeated measures are assessed over several time points and missing values need to be handled [51, 52]. All participant that are randomly assigned to one of the conditions are entered into the analysis (intent-to-treat = ITT-analysis).

Effect sizes will be calculated for imputed data (multiple imputations with 50 imputations) taking into account the dependence of data collected within participants to avoid the loss of power due to incomplete cases [53].

The number of patients experiencing a reliable change in each group will be reported giving an estimate on improvement/deterioration not attributable to chance taking into account the reliability of the measure [54]. For this calculation the standard error at the first point of measurement (T0) will be used, because it has not yet been influenced by any intervention.

Secondary analysis will include a per-protocol analysis (PPA - including all participants who adhered to the protocol), a repetition of the main analysis with outliers excluded, and a repetition of the analysis using the PHQ-9 results and a subgroup analysis, all using mixed models. In the subgroup analyses, possible moderating variables (e.g. gender, CBT-experience or frequency and length of logins) are going to be examined.

Changes in health-related quality of life (SF-12) will be analyzed, again, using mixed models, and differences in patient satisfaction (CSQ-8) will be confirmed using a *t*-test or a Mann-Whitney *U*-test, if normal distribution is violated.

An exploratory analysis of possible adverse events (INEP and protocols of the guidance calls) will be conducted. Frequencies of the occurrence of adverse events will be reported and compared between intervention group and control group using chi-sqaured tests.

Frequency and length of logins (according to self-rating) and the amount of guidance will be reported descriptively.

Analyses will be performed using an alpha level of .05, testing two-sided. All analyses will be conducted using SPSS [55] or R [56].

## Discussion

Because of its high prevalence and significant impact on many people’s lives, improvements and innovations in the treatment of depression should be one of the major concerns of public health systems. Despite the fact that a variety of effective treatments are available, depression is still undertreated [9].

Building on previous results indicating internet based interventions (IBIs) to be an effective treatment for depression, the results of the proposed study will further contribute to the understanding of IBIs as a supportive part of the treatment of depression. It represents the first randomized controlled trial on the iFightDepression® tool in its German version. It aims not only at demonstrating the effectiveness of the iFD® tool, but also at providing new insights into aspects of e-mental health research that are so far understudied, namely the specificity of the treatment effect compared to PMR as an active control condition, its continuity over a time course of 12 months and possible negative effects of iCBT.

In their consensus statement, Rozental et al. pointed out the importance of more in-depth studies on negative effects of iCBT [21]. In an individual patient data meta-analysis, Ebert and colleagues could show that the possibility of symptom deterioration in iCBT studies is comparable to that of psychotherapy studies and significantly smaller in the treatment arms compared to waitlist controls [19]. Still, there are other possible adverse events that patients might experience (e.g. feeling dependent on the program or stopping medication without a prior consultation with their physician). The present study will provide further insight into possible risks associated with iCBT solutions.

Furthermore, it will implement an active control condition as waitlist control designs might be insufficient to differentiate between effects of the active ingredients of a treatment and the induction of hope (often named an “unspecific effect” in psychotherapy research). It is known that the choice of the control condition has a larger impact on the effect size than the specific treatment [24]. Several aspects in IBI RCT studies might have the potential to induce hope in participants independently of the efficacy of the intervention, e.g. repeated contact with study personnel conducting the guidance and several assessments, as well as the potential subjective impression of participants of “doing something” (namely participating in this study on depression online interventions).

To reduce the effect of ‘hope induction’ and to have a more rigorous test of iCBT as an adjunct to the treatment of depressed patients, PMR is used as an active control condition. The condition was chosen to match the intervention in the time spent on the intervention and frequency of use. PMR is a well-accepted adjunct to the treatment of depression and, therefore, has the potential to induce hope. It does not, however, target specific symptoms of depression such as automated negative thoughts or reduced positive activities.

Through the use of a guidance protocol, we ensured that the amount of guidance is comparable in both intervention arms. In addition, the guidance protocol enables study assistants to match the length and content of guidance to that recommended for primary care guides of the iFD®-tool.

Still, there are some limitations of the proposed study that should be taken into account. First of all, the results cannot be generalized for iFightDepression® in other countries. Cultural aspects as well as previous knowledge and experience with CBT might play a role in the efficacy of the iFD® tool, so the results should be interpreted within the cultural background.

Also, even if the active control was chosen carefully as a credible and accepted self-management technique, it might be that participants still have a different preference for either the iFD® tool or for PMR. Since PMR has been widely used to support the treatment of depression, the iFD® tool could be preferred because it is a new and hope-inducing alternative. This problem is difficult to avoid, since a fraud treatment would be unethical. Possible preferences of participants should be checked through a comparison of the dropout rates and reasons between the intervention groups.

To our knowledge, the current study is one of the first to test iCBT for specific treatment effects (those effects that are caused by the content of the treatment and not by inducing hope) through comparing to an active control condition. And, although the choice of an active control condition may lead to substantially reduced effect sizes, this stricter examination might result in even stronger arguments for the use of iCBT solutions in depressed patients.

## Abbreviations

CBT: Cognitive Behavioural Therapy
CIDI: Composite International Diagnostic Interview
CSQ-8: Client Satisfaction Questionnaire
DF: German Depression Foundation
DSM-IV: Diagnostic and Statistical Manual of Mental Disorders
GP: General practitioner
IBIs: Internet based Interventions
iCBT: Internet based Cognitive Behavioral Therapy
IDS-C: Inventory of Depressive Symptomatology – clinician rated
IDS-SR: Inventory of Depressive Symptomatology – self-rating
iFD: iFightDepression
INEP: Inventory for the assessment of negative effects of psychotherapy
ITT: Intent to treat
M.I.N.I.: Mini International Neuropsychiatric Interview
MDD: Major Depressive Disorder
PHQ-9: Patient Health Questionnaire-9
PMR: Progressive muscle relaxation
PPA: Per-protocol analyses
RCT: Randomized controlled trial
SF-12: Short form 12
SKID-P: Structural Clinical Interview for DSM-IV
SOP: Standard Operating Procedures
TAU: Treatment as Usual
WL: Waitlist controls
ZUF-8: German version of the client satisfaction questionnaire
